# Characterization of Chestnut Tannins: Bioactive Compounds and Their Impact on Lamb Health

**DOI:** 10.3390/life14121556

**Published:** 2024-11-27

**Authors:** Bogdan Cekić, Jordan Marković, Vuk Maksimović, Dragana Ružić-Muslić, Nevena Maksimović, Ivan Ćosić, Krstina Zeljić Stojiljković

**Affiliations:** 1 Institute for Animal Husbandry, Belgrade-Zemun, Autoput 16, P.O. Box 23, 11080 Belgrade, Serbia; druzicmuslic@istocar.bg.ac.rs (D.R.-M.); nmaksimovic@istocar.bg.ac.rs (N.M.); icosic@istocar.bg.ac.rs (I.Ć.); kstojiljkovic@istocar.bg.ac.rs (K.Z.S.); 2 Institute for Forage Crops, Globoder, 37251 Kruševac, Serbia; jordan.markovic@ikbks.com; 3 Department of Life Sciences, University of Belgrade—Institute for Multidisciplinary Research, Kneza Višeslava 1, 11030 Belgrade, Serbia; maxivuk@imsi.bg.ac.rs

**Keywords:** ruminant nutrition, fattening lambs, blood metabolites, polyphenols, supplementation

## Abstract

The objective of the present study was to characterize the chestnut (*Castanea sativa* Mill.) tannin product, Farmatan Plus^®^ (Tanin Sevnica d.d., Sevnica, Slovenia), and to subsequently examine its effects on the blood metabolic parameters of fattening lambs, particularly in relation to their health status. Thirty lambs were randomly divided into three treatment groups: a control group without added tannin and two groups that received 9.46 g of the tannin product/kg of the diet dry matter (DM) and 18.87 g of the tannin product/kg of the diet DM. Metabolic parameters such as contents of total protein, globulin, urea, and liver enzymes (AST and GGT) were measured over a trial period of 60 days to evaluate the effects of tannin supplementation. This study represents the first in-depth characterization of Farmatan Plus^®^, demonstrating its richness in bioactive compounds such as vescalin and castalagin. The results showed no significant adverse effects on lamb health, with all parameters remaining within normal physiological ranges (*p* > 0.05). These results support the safe inclusion of chestnut tannins in the diet of lambs and underline their potential as a functional feed additive that can positively influence the health and growth performance of ruminants.

## 1. Introduction

Tannins are a diverse group of secondary metabolites that are widely distributed in plants [1]. Historically, they have been viewed as antinutritional due to their negative effects on animal health and productivity, such as reduced feed intake [2], lower fiber and sugar digestibility [3], weight loss [4], and toxicity [5]. High levels of tannins in feed, especially hydrolysable tannins (HT), can lead to enzymatic depolymerization in the rumen, resulting in health problems and, in extreme cases, death [6,7,8].

On the other hand, tannins have been shown to provide benefits such as improved feed conversion [9], higher average daily growth [10,11], antioxidative effects and better meat quality [12], improved reproductive parameters [13], higher milk production and quality [14], as well as antiparasitic [15], antidiarrheal effects [16], and even reduce methanogenesis [17].

The effects of tannins on ruminants are influenced by several factors, including the plant species, specific types of tannins such as condensed tannins (CT) and HT, and their concentration in the diet since they differ structurally and functionally [18]. CT, which consists of flavonoid units linked by carbon bonds, resists hydrolysis and can reduce the bioavailability of nutrients, especially in monogastric [19]. In ruminants, CT can improve protein utilization by limiting degradation in the rumen, although high levels may impair digestibility [20]. HT, whose carbohydrate core is linked to phenolic acids, is more water soluble and is hydrolyzed in the rumen, releasing phenolic acids such as gallic acid, which can have different effects depending on their concentration [21]. In addition, animal species, age, physiological condition, and feed composition influence the effects of tannins [22]. Depending on these variables, tannins can have a range of effects, from improving protein utilization and amino acid availability to potentially negative impacts [23].

Given tannins heterogeneity, a detailed analysis of their sources is crucial. The usual classification into CT and HT is insufficient [24] as many plant species contain both [19]. Sweet chestnuts (*Castanea sativa* Mill.) contain both types, although the proportion of HT is higher than that of CT [21]. Still, the tannin content and quality of extracts vary depending on the age of the plant, growing location, soil type, and extraction method [25,26]. Chestnut tannins include compounds such as vescalin, castalagin, gallic acid, ellagic acid, and others [27,28,29], and they may also contain flavonoids such as rutin, quercetin, and apigenin [30].

Extraction methods can alter tannin compounds and product quality. For instance, heating in an aqueous solution can convert castalagin to vescalagin, while acid hydrolysis converts castalagin to ellagic acid and castalin [26,31]. Methods such as the determination of total polyphenols using the Folin–Ciocalteu method and the use of HPLC to analyze individual tannin compounds have been developed to address this complexity [32]. However, the discrepancy in the description of the source of tannin used is still present in the literature.

Despite extensive research, the effects of tannins remain inconsistent due to their complexity. The literature increasingly indicates that the effects of tannins should be evaluated based on the plant species, the tannin content in the diet, the general diet composition, and the species and age of animals. The aim of this study is, therefore, to characterize the chestnut tannin product used and to examine how different amounts of it in the diet affect metabolic parameters, reflecting the health status of fattening lambs.

## 2. Materials and Methods

### 2.1. Animals and Experimental Design

The trial was conducted at the Institute for Animal Husbandry in Belgrade, Serbia (44°50′20.66″ N, 20°17′07.09″ E), in strict compliance with the usual ethical standards and approved by the Veterinary Administration of the Ministry of Agriculture, Forestry and Water Management of Republic of Serbia (approval number 323-07-05054/2018-05).

This study included 30 MIS lambs (meaty breed formed by crossbreeding of local native breed Pirot Pramenka, Merinolandschaf, and Ile de France [33]) enrolled in the experiment after weaning (at 60 ± 3 days of age) and blocked by body weight (BW); three treatment groups were formed with 10 male lambs in each group, with average BW of 20.55 kg and body condition score (BCS) of 3, and dry matter intake (DMI) 1.18 kg, without statistical significance between groups (*p* > 0.05). All three groups of lambs were divided into five separate pens (2 lambs per pen) using movable partitions. During the experiment, lambs were housed in an enclosed pen with a wheat straw deep mat and had *ad libitum* access to water. At birth, the lambs were injected with selenium and vitamin E. One month after birth, a vaccine against clostridial infections was administered, with a booster vaccination 14 days after the first dose. At the age of three months, the lambs were vaccinated for active immunization against bluetongue disease.

The experiment was designed as a single-factor trial with three treatments to investigate the effect of chestnut tannins on metabolic blood parameters in lambs. Three groups were formed depending on the amount of calculated chestnut tannin product in the diet:

C—control (no added tannins),

T1, which received 9.46 g of tannin product per kg DM of diet (15 g of tannin product per kg of concentrate mixture—13.5 g of tannin product per day), and

T2, which received 18.87 g of tannin product per kg DM of diet (30 g of the tannin product per kg of concentrate mixture—27 g of tannin product per day).

The experiment lasted 67 days, including 7 days for adaptation and 60 days for data collection. The diet for the lambs consisted of a roughage (0.7 kg) and a concentrate portion (0.9 kg) and differed in the amount of chestnut tannin product added (0 g, 15 g, and 30 g/kg concentrate mixture). The roughage portion of the diet consisted of alfalfa hay obtained from the arable land of the Institute for Animal Husbandry and was harvested during the second cut at the beginning of the flowering period. The average harvest height for alfalfa was about 50 cm, with a cutting height of about 10 cm. Standard field procedures included turning the cut biomass, baling the hay, and storing the bales, which weighed an average of 15 kg each. The concentrate mixture was produced in the Institute of Animal Husbandry’s feed production plant. It was in milled form and consisted of maize, sunflower meal, wheat bran, salt, monocalcium phosphate, chalk, and a vitamin-mineral premix. A used vitamin-mineral premix included monocalcium phosphate, calcium carbonate, sodium chloride, trace elements, vitamins, organically bound selenium, live yeast cells, mycotoxin binder, antioxidants, and corn flour as a carrier for the premix. The nutritive value of the diet was estimated using the CVB 2016 nutritional system [34]. The ingredients (feeds), chemical composition, and nutritive value of the diet are shown in Table 1.

### 2.2. Extraction of Tannins, Chemical Analysis, and Characterization of Phenolic Compounds in Used Tannin Product

The tannin source used was a tannin product isolated from the wood of the sweet chestnut (*Castanea sativa* Mill.), which is commercially available under the name Farmatan Plus^®^ and is manufactured by Tanin Sevnica (Tanin Sevnica d.d., Hermanova 1, 8290 Sevnica, Slovenia). According to the manufacturer, the product was obtained from locally grown trees in Slovenia. After de-barking, grinding bark-free chestnut wood, and hot water extraction, followed by filtration, concentration, and drying of the extract, the product consisted of 73 ± 2% chestnut extract and 0.1% sweetener (stevia extract). Organoleptically, the product appears as an amorphous, fine powder with a rich brown hue, accompanied by a pronounced woody aroma and a distinctly astringent taste.

In order to analyze the composition of Farmatan Plus^®^, representative samples from the used batch were collected and processed into a uniform sample using the quartering method. In the laboratory, all analyses were carried out in triplicates.

For the extraction, approximately 2.00 g of the tannin product was weighed, and 20 mL of the extraction solution (ethanol:water:HCl = 80:19:1) was added. After incubation at room temperature for 2 h, it was decanted, and the solid residue was extracted twice more with 20 mL and 10 mL of the extraction solution. The filtrate obtained by filtering the collected extracts through a Büchner funnel was transferred to a 50 mL volumetric flask and diluted to the mark with the extraction solvent. The resulting extract was stored in refrigerated conditions and used for the analysis of total polyphenols, flavonoids, and condensed tannins.

The concentration of the individual phenolic compounds in the chestnut tannin product was determined using reversed-phase high-performance liquid chromatography with mass spectrometry (RP-HPLC-MS). For this analysis, an additional extract was prepared by extracting the soluble tannins of the product with methanol. The extraction was carried out overnight in the absence of light and at a temperature of about 4 °C. The extract was diluted 25-fold in methanol and filtered through a 0.22-micron nylon syringe filter.

Samples were injected into an HPLC system (Waters, Milford, MA, USA) consisting of pumps, a thermostat, and an autoinjector connected to a Waters 2996 diode array detector and an EMD 1000 quadrupole detector with an electron spray ionization (ESI) probe (Waters, Milford, MA, USA). The phenolic compounds were separated using a Symmetry C-18 RP column 125 mm × 4.6 mm size packed with 5 µm diameter particles (Waters, Milford, MA, USA) and connected to a corresponding precolumn.

Two mobile phases, A (0.1% formic acid solution) and B (acetonitrile), were used at a flow rate of 1 mL/min in the following gradient profile: 0.0–20.0 min 10% B, 2.0–30.0 min from 10% to 20% B, 30.0–35.0 min from 40% to 50% B, 35.0–45.0 min from 50% to 70%, then 10% B for 15 min.

A post-column flow splitter (ASI, Richmond, VA, USA) with a ratio of 5/1 was used to achieve an optimal flow rate (0.2 mL/min) of the mobile phase for the ESI probe. For LC/MS analysis, the signals of each component were detected in negative scanning mode (100–1000 *m*/*z*) with the following ESI source parameters: capillary voltage—3.0 kV; cone voltage—35 V; extractor and RF lens voltages were 3.0 V and 0.2 V, respectively. The source temperature and desolvation temperature were 130 °C and 400 °C, respectively, in an N_2_ flow of 500 L/h. In the absence of specific standards, compounds are ranked on a scale of 1 to 5, with each level representing increasing concentrations, from absence to predominant presence.

Data acquisition, processing, and spectral analysis to confirm the peaks of specific compounds were performed using Waters Empower™ software, version 2 (Waters, Milford, MA, USA). The classification of most compounds was based on the PubChem open database of chemical compounds [35], with the exception of cretanine, whose classification was based on Oracz et al. [36] description. The defined classes include HT, phenolic acids (PAC), phenolic glucosides (PG), flavonoids (FL), and phenolic aldehydes (PAL).

The concentration of total polyphenols was determined spectrophotometrically (Spekol 1300, Analytik Jena GmbH, Jena, Germany), using the Folin–Ciocalteu method [37] the concentration of flavonoids using the method described by Makkar [32] and the content of CT using method described by Nakamura et al. [38]. These methods were applied to all feedstuffs included in the diet, in addition to the tannin product. To analyze the feed ingredients, representative composite samples were collected from hay bales and concentrate mixtures, and average samples were obtained by quartering. All feed samples were analyzed in triplicate.

In order to analyze the chemical composition of the tannin product used, the representative sample was obtained using the quartering method, and all analyses were carried out in triplicates. Dry matter was determined by drying the sample in a controlled environment. Total nitrogen and crude protein were analyzed using the Kjeldahl method using a Gerhardt Vapodest 50S instrument (Gerhardt GmbH & Co., Königswinter, Germany), with crude protein calculated from the nitrogen content. Crude fat was measured using the Soxhlet extraction method with a Gerhardt Soxtherm device (Gerhardt GmbH & Co., KG, Königswinter, Germany) according to AOAC 920.39. The crude fiber was determined by successive hydrolysis with dilute H_2_SO_4_ and NaOH according to AOAC 978.10. Crude ash was determined by incineration of the sample at 550–600 °C according to AOAC 942.05.

### 2.3. Blood Sampling and Analysis

On the first and last day of the experiment, blood was taken from the lambs for biochemical tests via venipuncture of the jugular vein (*v. jugularis*). The blood samples were collected in standard Vacutainer tubes (BD Vacutainer^®^ SST II Advance with a volume of 3.5 mL; BD Vacutainer^®^ K2E 3.6 mg with a volume of 2.0 mL; BD Vacutainer^®^ NaF 3.0 mg, Na_2_EDTA 6.0 mg with a volume of 2.0 mL).

The blood samples were stored in a cold chain at a temperature of 2 to 8 °C, and the analyses were performed on the same day the samples were taken. The blood samples for serum extraction were left at room temperature for natural coagulation until transportation to the laboratory (within 4 h). They were then centrifuged at 1000 rpm for 20 min to separate the serum. After serum separation, the samples were stored at 4 °C until they were analyzed on the same day.

The biochemical parameters included total proteins, albumin, globulins, urea, creatinine, glucose, total bilirubin, transaminases (and), creatine kinase—CK, cholesterol, triglycerides, calcium, phosphorus, and magnesium.

The analyses were performed using an Olympus AU 400 device (Olympus Corp., Center Valley, PA, USA), and the methods and reference values were defined according to Baumgartner and Wittek [39]. The following methods were used for the observed parameters: total proteins—biuret method; albumin—bromocresol green; globulins—calculation method (subtraction of total proteins and albumin content), urea—urease/glutamate dehydrogenase (GLDH); creatinine—Jaffe IDMS traceable (method A); glucose—hexokinase–glucose-6-phosphate dehydrogenase (HK G6P-DH); total bilirubin—diazonium ion (DPD); aspartate transferase (AST)—modified method without P-5-P; gamma-glutamyl transferase (GGT) and creatine kinase (CK)—IFCC; cholesterol—liquid cholesterol (CHO-POD); triglycerides—coupled enzyme reaction; calcium—arsenazo method; phosphorus—UV molybdate; magnesium—Xylidyl Blue-I method.

### 2.4. Statistical Analysis

The experiment was designed as a single-factorial experiment with three treatments. The differences between the control group and the treatments were analyzed using a one-way analysis of variance (ANOVA) with IBM SPSS Statistics software, version 20 (SPSS Inc., Chicago, IL, USA) to evaluate the effects of the different tannin levels in the lambs’ diet. Significant differences between the groups were determined using Tukey’s Honest Significant Difference (HSD) post hoc test. The results are given as mean values ± standard deviation, with statistical significance set at *p* < 0.05.

## 3. Results

### 3.1. Characterization of Used Tannin Product

As shown in Table 2, the chestnut tannin product used had a high concentration of polyphenols (36.14 g/100 g DM) and flavonoids (17.01 g/100 g DM). It also contained moderate amounts of crude protein (13.75 g/kg DM) and crude ash (19.40 g/kg DM), reflecting its potential as a bioactive feed additive.

In addition to the high polyphenol and tannin content, the product had low levels of crude fat (1.20 g/kg DM) and crude fiber (0.50 g/kg DM), making it a concentrated source of bioactive compounds with minimal high-energy components. The balanced crude protein and nitrogen content support its suitability as a functional feed additive, potentially enhancing both the health and productivity of livestock without compromising metabolic function.

The chestnut tannin product contains a variety of bioactive compounds (Table 3), especially HT, PAC, and FL. The Supplementary Figure (Figure S1) presents HPLC chromatograms of the most abundant compounds in Farmatan Plus^®^ recorded in single ion recording mode, with each inset displaying a characteristic MS spectrum for the peak observed at the corresponding *m*/*z* value.

The most important HT compounds include vescalagin and castalagin, both of which are present in high amounts and have a molecular weight of 934 g/mol. Phenolic acids, such as gallic acid and ellagic acid, as well as digalloyl glucose, also play an important role and contribute to the antioxidant properties of the product. Flavonoids such as astragalin and kaempferol coumaroyl hexoside are present in smaller amounts but increase the functional potential of this product as a natural feed additive. In addition to the main groups of tannins, other notable compounds such as protocatechuic acid, vanillic acid, and syringic acid were also found to contribute to the phenolic profile of the product. The presence of aldehydes such as vanillin and sinapaldehyde, albeit in lower concentrations, adds to the complexity of the tannin extract.

The combination of these bioactive compounds, including both HT and PAC, underscores the product’s potential as a functional additive with antioxidant and anti-inflammatory properties suitable for improving ruminant health and nutrition.

### 3.2. Blood Metabolic Profile

At the beginning of the experiment (Table 4), the blood parameters differed between the lamb groups, although not significantly (*p* > 0.05). Nevertheless, deviations were found in the comparison with the reference values. Protein (57.59–58.13 g/L) and globulin (24.71–26.59 g/L) levels were below the physiological range, indicating possible hypoproteinemia. In contrast, urea levels were higher than normal, suggesting inefficient protein utilization and possible nutrient stress during weaning. Creatinine levels were in the normal range in the control group but were slightly lower in the tannin groups. Elevated GGT and CK levels indicated muscle stress and handling-related fatigue, while triglycerides, calcium, phosphorus, and magnesium were generally within or near normal levels, with slight variations likely due to individual metabolic differences and mild dehydration.

At the end of the experiment, the blood parameters (see Table 4) also showed some divergence from the reference values, but no statistically significant differences were found between the groups (*p* > 0.05), indicating that the chestnut tannins at both doses (9.46 g product/kg DM od diet and 18.87 g product/kg DM of diet) had no negative effects on the health of the lambs. Total protein and albumin levels remained within the normal range, although globulin levels were slightly lower in the tannin groups. Urea levels were within the reference range, indicating optimal protein and energy balance. Creatinine levels remained below physiological levels, suggesting no impairment of renal function. Glucose concentrations were normal in the tannin groups, while they were slightly elevated in the control group, and bilirubin remained within the reference ranges, indicating normal liver function. Elevated AST and GGT levels, which occurred in all groups, were probably due to stress or muscle fatigue and not to tannin ingestion. CK levels were high in all groups, indicating stress, but were slightly lower in the tannin-fed animals. Cholesterol and triglyceride levels showed no significant differences, indicating that chestnut tannins do not significantly affect fat metabolism.

## 4. Discussion

### 4.1. Specificities of the Used Tannin Product

Sweet chestnut (*Castanea sativa* Mill.) is a significant commercial source of hydrolysable tannins but also contains condensed tannins [27,40]. In our analysis, consistency with the literature data on the phenolic compounds present in the preparation (Table 2 and Table 3) was confirmed, aligning with the findings of several authors [27,41,42,43].

By using the HPLC method, 11 HT compounds were detected, including gallic acid, ellagic acid, vescalagin, castalagin, and roburin A–E, which collectively accounted for 36.3% of the preparation [40]. While some of these compounds (e.g., roburin, pedunculagin, O-galloyl-castalagin) could not be detected in our study, the polyphenolic profile consisted mostly of HT, supporting previous findings. Chestnut tannin products are a valuable bioactive source, mainly due to the antioxidant and anti-inflammatory properties of gallic acid, which may contribute to the antimicrobial and antioxidant effects of chestnut products in protecting the gastrointestinal, nervous, and cardiovascular systems [44].

The comparison of tannin preparations and products in different studies is challenging due to the different tannin sources and methods of analysis. Some studies only report the source without specifying the tannin content [9,10,45,46], while others rely on manufacturer specifications [6,47,48,49,50,51,52,53,54,55], which makes direct comparison difficult.

Differences in the methods used to determine CT and HT make comparisons between the studies difficult. For example, the butanol–HCl method was used to measure CT in chestnut and mimosa tannins [56,57], with results expressed as a percentage of the preparation. In contrast, the vanillin method [38] was used in this study, and the results were expressed as catechin equivalents (g/100 g DM). Fernandes et al. [58] calculated the HT content by subtracting CT from the total tannins.

Studies using the same tannin product as in our study offer only limited comparability. Although many authors reported the use of Farmatan^®^ [59,60,61,62,63,64], they did not report its chemical composition. Some studies only reported the tannin content without further details. According to the literature, two groups of studies can be distinguished: one reporting a tannin content of 55% [65,66] and another reporting 75% [19,26,67,68]. However, the manufacturer’s specification states that the product contains 73 ± 2% chestnut extract. This variability in the data makes the comparisons between the studies even more complex.

The literature also references chestnut tannin preparations from the same manufacturer under a different name (Tanimil SCC^®^). In the studies [69,70], it was reported that this preparation contains 40% tannins, while in [71], its use was only mentioned without specifying the composition. If the term “tannins” in these studies refers to total polyphenols, the used product in our study is comparable as it contains 361.40 g GAE/kg DM. However, terminological inconsistencies—some authors equate tannins with total polyphenols—make a direct comparison uncertain.

The total polyphenol content given here agrees with the values reported by other authors, who found polyphenol values between 0.67 and 569.9 g GAE/kg in chestnut samples using the Folin–Ciocalteu method [41]. In the other study, 436 g GAE/kg was determined using the same product (Farmatan^®^) and the same method [72]. The discrepancy in these results is due to differences in raw materials (especially the plant material used) and production methods. Factors such as the origin of the chestnut, the age of the tree, and the tissue type influence the tannin content [26,73], as the tannin distribution varies within the plant tissue. Insoluble phenols are located in the cell walls, while soluble phenols are located in the vacuoles, with the outer layers having a higher phenolic content [73].

To enable more accurate comparisons of chestnut tannin preparations, standardized methods for the analysis of tannin content and a clear indication of the chemical composition are essential. This study is beneficial as it applies methods for the analysis of polyphenol content and allows for a clear identification of the phenolics in the product. Accounting for differences in extraction methods, plant origin, and tissue type will improve consistency between studies and better highlight the bioactive potential of chestnut tannins.

### 4.2. Blood Parameters

At the beginning of the study (Table 4), differences were found in the blood parameters of the lambs, but these were not statistically significant (*p* > 0.05). All lambs had been reared in a similar manner, spending time with their mothers (before weaning) and being fed identical concentrate mixtures and alfalfa hay. The elevated values of several parameters indicated stress during group housing and suboptimal water intake, probably due to poor adaptation to automatic drinkers.

The concentrations of total protein (57.59–58.13 g/L) and globulin (24.71–26.59 g/L) were below physiological values (59.00–78.00 g/L for total protein and 32.00–50.00 g/L for globulin), indicating possible hypoproteinemia. This indicates a reduced protein supply to the small intestine, possibly due to reduced microbial protein synthesis in the intestinal wall or other contributing factors [74]. Protein metabolism involves degradation by proteases, releasing amino acids that are absorbed and contribute to blood protein levels. Increased microbial protein synthesis in the rumen normally increases protein levels in the blood [71], but this effect was not observed at the start of the study. The most common cause of hypoproteinemia is inadequate protein intake, which can result in reduced globulin and other protein synthesis [75]. While various factors (parasitic infections [15], liver or kidney issues [20,24]) could contribute, reduced feed or protein intake is a likely factor in this case.

In contrast to globulins and total proteins, urea concentrations were higher than the reference values in all groups. Elevated urea levels may indicate inefficient protein utilization due to an imbalance between the intake of degradable protein and the microbial demand in the rumen, resulting in increased liver urea formation, which is energy intensive [76]. Ammonia from protein degradation in the rumen is absorbed and detoxified to urea in the liver, a process that is influenced by energy and protein supply. Reduced energy intake, often due to carbohydrate deficiencies, may limit microbial protein synthesis and increase blood urea levels [51]. This suggests that prior to the trial, during the weaning phase, lambs may not have fully utilized nutrients due to stress and reduced milk intake from sheep fed a low-energy diet, contributing to higher urea and lower protein levels [77].

Creatinine levels in the control group (77.72 µmol/L) were within the reference values, while they were slightly lower in the tannin groups (74.70 and 74.06 µmol/L for T1 and T2). Creatinine, a by-product of muscle metabolism, remains relatively stable under normal conditions. Lower levels can be caused by factors such as dietary changes, malnutrition, or dehydration [78]. Since all lambs were housed together prior to this study, stress, reduced water intake, and mild dehydration likely contributed to these lower creatinine concentrations.

Transaminases, which enable amino acid and α-keto acid reactions, are important indicators of liver function [52]. In this study, AST levels were elevated only in the T1 group, while they were within the reference ranges in the control and T2 groups. The elevated AST level in the T1 group probably reflects stress or muscle fatigue, as AST is also present in muscle tissue. Meanwhile, GGT levels were elevated in all groups, and given its role in glutathione metabolism and liver function, this increase suggests stress rather than liver injury, likely due to the stress associated with weaning and rearing the lambs [78].

CK levels were above reference values in all groups, consistent with their role as markers of muscle stress or injury. Elevated CK levels are common in young, growing animals and those exposed to physical activity or stress [79]. This increase indicates that the lambs have experienced muscle fatigue, likely due to handling and stress.

Triglyceride levels were normal in the control and T2 groups but slightly elevated in the T1 group (0.34 mmol/L). Triglycerides, which are important for energy metabolism, are mainly processed in the liver [52]. The variations in triglyceride levels could be due to individual differences in metabolism rather than diet problems, as all lambs were kept under similar conditions prior to this study.

Calcium levels were within the reference values in all groups, while phosphorus and magnesium were elevated. Elevated phosphorus, which is often associated with dehydration, is usually well utilized by ruminants as the microorganisms in the rumen break down phytate [80]. Increased magnesium, an important enzyme activator in metabolism, is less common and may be due to increased dietary intake, improved absorption, or mild dehydration [39].

The initial metabolic profile showed differences between the groups, but these were not statistically significant (*p* > 0.05). All lambs were reared in a similar manner, with a concentrated mixture of alfalfa hay and limited time with their mothers. The increased parameters indicate stress due to group housing and suboptimal water adaptation due to unfamiliarity with the automatic drinkers.

The final health assessment carried out on the last day of the trial (Table 4) provides further insight into the effects of chestnut tannins on the lambs. Analysis of blood parameters revealed some deviations from reference values, but no statistically significant differences were found between groups (*p* > 0.05). This indicates that chestnut tannins at a dosage of 9.46 g/kg DM (T1) and 18.87 g/kg DM (T2) had no negative effect on the health or welfare of the lambs.

Compared to baseline values, total protein, and albumin concentrations remained within the reference ranges in all groups despite lower globulin levels in T1 and T2 and close to the lower limit in the control group. This result is consistent with the findings of [47], where no significant changes in total protein, albumin, or globulin concentrations were found when chestnut and quebracho tannins were included in the diet of ruminants. This agreement suggests that tannins, whether from chestnut or quebracho, do not significantly affect the balance of these proteins. When investigating the effects of mimosa tannins, no significant effects on the metabolic profiles of sheep were found either [81]. However, a tendency towards higher protein concentrations was found, which may be due to the anthelmintic effect of the tannins. While our study did not specifically investigate such effects, the maintenance of total protein levels within the reference range despite lower globulin levels may indicate improved protein utilization, possibly related to a similar mechanism. However, an increase in globulin and total protein levels in lambs was observed with tannins and cellulase, especially in the group receiving both tannins and cellulase, suggesting a possible synergistic effect [82]. While cellulase was not used in our study, the increased protein utilization observed may indicate a similar interaction where tannins alone improve protein availability, although not to the extent seen in combined treatments as in the mentioned study. This suggests that while tannins may improve protein utilization, their effect may be enhanced when combined with other agents, such as cellulase—a finding worth exploring in future research.

In contrast to baseline values, urea concentrations were within the reference range in all groups, indicating that the diet provided an optimal protein and energy balance without adverse effects of tannins. This is consistent with the other authors, where no significant differences between the tannin-treated groups and the control groups were found [83]. Results obtained in this study support the idea that tannins in adequate amounts do not negatively affect protein metabolism.

Creatinine concentrations remained below physiological levels in all groups, which is consistent with trends observed since the beginning of the experiment. The lack of significant differences suggests that chestnut tannins do not impair renal function, a conclusion supported by other authors who also found no significant differences in creatinine levels between tannin and control groups [6]. Similarly, peanut shell tannins were found to have no effect on creatinine levels [84], suggesting that various types of tannins in low to moderate amounts in the diet have minimal effects on kidney function.

Glucose concentrations in the T1 and T2 groups remained within the physiological range, with slightly elevated levels in the control group, although not statistically significant. These results are consistent with those of other authors [85], who found no significant differences between the control and tannin groups. Conversely, an increased glucose level was observed with a higher intake of peanut shell tannins, which was attributed to increased production of propionic acid in the rumen, a known precursor of glucose in the hepatocytes [84]. The effect of tannins on elevated glucose levels can also be explained in other ways. One possible mode of action of tannins is that they reduce ammonia production, possibly lowering the energy requirements of bacteria and increasing the availability of glucose [77]. The increased glucose concentration could also be due to the fact that tannins form complexes that protect proteins from degradation and thus promote amino acid uptake and glucose synthesis through gluconeogenesis [86]. Although these mechanisms were not observed in our study, the results suggest that tannins may influence glucose metabolism under certain conditions, warranting further investigation of their effects on energy utilization.

Bilirubin concentrations remained within the reference range, suggesting normal liver function and no significant stress in the tannin-fed animals, which is consistent with results in [71]. This also indicates adequate energy status, as bilirubin is often used to assess energy adequacy [87]. In our study, this is consistent with the conclusion that the diet provided sufficient energy, and the intake of tannin had no negative effects.

AST transaminase levels were above physiological norms in all groups, although no significant differences were found between groups (*p* > 0.05). Considering that the control group also had elevated AST levels, it is unlikely that the tannins were a contributing factor. Instead, other factors, such as stress or muscle fatigue, could be responsible for these increases [88]. GGT transaminase levels were also elevated but without significant differences, which also suggests that the tannins have no specific effect on liver function. This is consistent with results in [89], where no significant changes in liver enzymes with tannin ingestion were found, suggesting no negative effects on the liver. Similarly, no significant differences in liver enzyme concentrations with different types of tannins were reported [90], supporting our findings. However, higher GGT and AST levels in tannin-fed animals were observed [6], which is in contrast to our results and may be due to differences in tannin sources or dosages.

CK levels were elevated in all groups, indicating stress or muscle fatigue, as CK is a marker of muscle injury [79]. The elevated CK levels probably reflect stress during handling and venipuncture. Although there were no significant differences between groups, the tannin-fed animals showed a trend towards lower CK concentrations compared to the control. It was reported that chestnut tannins reduced CK levels in lambs [91], suggesting a potential stress-reducing effect of tannins, which our results indicate, but further studies are needed to confirm.

Cholesterol levels remained within the physiological range in all groups, which is consistent with the results that found no significant differences between tannin-fed and control animals [92]. However, our findings differ from those of other studies, where increased cholesterol levels were observed with a higher intake of peanut shell tannins [84]. These differences could be due to the different types of tannins—HT from chestnuts in our study vs. CT from peanut shells—as well as differences in animal species (goats vs. lambs) and ration composition.

Triglyceride levels were slightly elevated in the T2 group but did not differ significantly from the control and T1 groups (*p* > 0.05). This is consistent with other authors [93], and no significant effects of chestnut tannins on triglycerides were observed. This suggests that chestnut tannins do not significantly alter lipid metabolism in lambs.

Our results confirm that chestnut tannins do not negatively affect lamb health, emphasizing their potential benefits for ruminant nutrition, and are consistent with the findings of other studies [52,94].

While our study focused on chestnut tannins, it is important to compare these findings with research on other sources of tannins to fully understand their role in ruminant nutrition. Studies on quebracho tannins have shown positive health effects on both sheep [47] and fattening lambs [95,96], although quebracho tannins contain mainly CT, as opposed to chestnut tannins, which are rich in HT. These differences in composition may affect digestibility and interaction with rumen microbes [21] while simultaneously influencing nitrogen retention [97].

Mimosa tannins, another condensed tannin source, have been shown to improve nitrogen retention in lambs [46,58], suggesting that different tannin structures may have similar benefits for protein metabolism.

In addition, oak tannins, as well as chestnut tannins, are mainly hydrolysable and have been studied for their astringent effect, which can reduce feed intake [24]. However, chestnut tannins appear to have milder effects on feed intake and may provide a balance between health benefits and palatability.

These comparisons show that while different sources of tannin can have comparable effects on animal health, conclusions must take into account factors such as the source of tannin, the structure, the form (native or extracted), the concentration in the diets, and the species and age of the animals.

## 5. Conclusions

This study shows that chestnut tannins, especially supplemented with Farmatan Plus^®^, can be safely incorporated into the diet of lambs without adverse health effects. In all treatment groups, key blood metabolic parameters—including total protein, globulin, and urea—remained within normal physiological ranges, suggesting that chestnut tannins do not negatively affect metabolism or organ function in lambs. Although liver enzyme levels were elevated, the lack of a significant difference between the control and treatment groups rules out the harmful effect of chestnut tannins.

The significance of this study lies also in the fact that the tannin product Farmatan Plus^®^ has been thoroughly characterized for the first time as an additive in ruminant nutrition. Its composition and bioactive compounds were highlighted, strengthening its potential as a safe and effective functional feed additive for ruminant nutrition. Characterization of the tannin product revealed that it is rich in bioactive compounds. The results suggest that chestnut tannins are a promising functional feed additive and can serve as a natural alternative to synthetic feed additives in ruminant nutrition.

However, some limitations are present. The relatively small sample size, the 60-day duration of the trial, the controlled experimental conditions, and the use of a specific tannin product (Farmatan Plus^®^) may limit the generalizability of the results. Larger, long-term studies under real-life conditions with different sources of tannin are needed to provide more definitive results, to fully understand their effects, and to assess possible delayed effects on the health and growth of lambs.

## Figures and Tables

**Table 1:** Ingredients, chemical composition, and nutritive value of diets.

Diet	Treatment *		
Ingredient	C	T1	T2
Alfalfa hay, g/day	700.00	700.00	700.00
Maize, g/day	479.70	472.50	465.30
Sunflower meal, g/day	281.70	277.47	273.24
Wheat bran, g/day	108.00	106.38	104.76
Salt, g/day	18.00	17.73	17.46
Monocalcium phosphate, g/day	2.70	2.70	2.61
Chalk, g/day	5.40	5.31	5.22
Vitamin mineral premix, g/day	4.50	4.41	4.41
Tannin product, g/day	0.00	13.50	27.00
Total, g/day	1600.00	1600.00	1600.00
Chemical composition, g/kg DM **			
Dry matter, g/kg diet	892.77	891.87	894.06
Crude protein	185.10	185.41	182.20
Crude fat	29.91	29.23	30.71
Crude fibre	182.76	180.16	178.25
Crude ash	67.64	64.73	66.90
NDF	329.04	316.31	313.09
ADF	231.62	232.24	232.86
Calcium	10.33	8.97	9.78
Phosphorus (total)	5.80	5.86	5.74
Total polyphenols g GAE/100 g DM	5.73	10.60	15.49
Flavonoids g CAE/100 g DM	3.72	6.02	8.31
Condensed tannins g CAE/100 g DM	0.41	0.59	0.77
Nutritive value, according to [29] ***			
NEmeat, MJ/day	9.96	9.86	9.75
Metabolic protein, g/day	118.31	117.21	116.11
bRP, g/day	14.91	15.08	15.25

* C—group without added tannins; T1—group that received 9.46 g of tannin product/kg DM of the diet; T2—group that received 18.87 g of tannin product/kg DM of the diet. ** NDF—neutral detergent fiber; ADF—acid detergent fiber; Total polyphenols g GAE/100 g DM—grams of gallic acid equivalent in 100 g of dry matter; Flavonoids and Condensed tannins expressed in g CAE/100 g DM—grams of catechin equivalent in 100 g of dry matter. *** NEmeat—net energy for growth; bRP—balance of degradable proteins.

**Table 2:** Chemical analysis of Farmatan Plus^®^.

Parameters *	Mean Value ± Standard Deviation
Dry matter, g/kg	898.30 ± 7.22
Total nitrogen, g/kg DM	2.20 ± 0.11
Crude protein, g/kg DM	13.75 ± 0.67
Crude fat, g/kg DM	1.20 ± 0.06
Crude fiber, g/kg DM	0.50 ± 0.03
Crude ash, g/kg DM	19.40 ± 0.79
Total polyphenols, g GAE/100 g DM	36.14 ± 1.78
Flavonoids, g CAE/100 g DM	17.01 ± 0.84
Condensed tannins, g CAE/100 g DM	1.34 ± 0.06

* DM—dry matter; Total polyphenols g GAE/100 g DM—grams of gallic acid equivalent in 100 g of dry matter; Flavonoids and Condensed tannins expressed in g CAE/100 g DM—grams of catechin equivalent in 100 g of dry matter.

**Table 3:** Characterization of phenolics in Farmatan Plus^®^.

No.	Compound	λmax *	MW (g/mol) **	Presence	Class ***
1	Vescalagin	224, 276	934	5	HT
2	Castalagin	224, 276	934	5	HT
3	Gallic acid	272	170	5	PAC
4	Ellagic acid	254	302	5	PAC
5	Dehydrated tergallagic-C-glucoside	250, 374	614	5	PG
6	Digalloyl glucose	274	484	5	PAC
7	Monogalloyl glucose I	274	332	4	PAC
8	Trigalloyl glucose	276	636	4	PAC
9	Cretanin	280	470	4	PG
10	Tetragalloyl glucose	276	788	3	HT
11	Vescalin	230, 280	632	2	HT
12	Castalin	230, 280	632	2	HT
13	Monogalloyl glucose II	274	332	2	HT
14	Ellagic acid—glucopyranoside	280	552	2	HT
15	Pentagalloyl glucose	274	940	2	HT
16	Protocatechuic acid	297, 258	154	2	PAC
17	Vanillic acid	260, 292	168	2	PAC
18	Syringic acid	274	198	2	PAC
19	Astragalin	280	448	2	FL
20	Kaempferol coumaroyl hexoside	280	594	2	FL
21	Protocatechualdehyde	280, 310	138	1	PAL
22	Vanillin	280, 312	152	1	PAL
23	Conifer aldehyde	290, 322	178	1	FL
24	Sinapaldehyde	300, 338	208	1	FL

* λmax—maximal wavelength; ** MW—molecular weight, expressed in g/mol; *** HT—hydrolysable tannins, PAC—phenolic acid, PG—phenolic glycoside, FL—flavonoid, PAL—phenolic aldehyde.

**Table 4:** Blood metabolic parameters at the beginning and the end of the trial (±standard deviation).

Par.*	Prot. g/L	Alb. g/L	Glob. g/L	Urea mmol/L	Creat. µmol/L	Gluc. mmol/L	Bilir. µmol/L	AST IU/L	GGT IU/L	CK mmol/L	Chol. mmol/L	Trig. mmol/L	Ca mmol/L	P mmol/L	Mg mmol/L
Group **															
Ref ***	59.00–78.00	27.00–37.00	32.00–50.00	3.70–9.30	75.80–174.30	2.40–4.5	0.70–8.60	49.00–123.30	19.60–44.10	7.70–101.00	1.10–2.30	0.20–0.30	2.30–2.90	1.30–2.40	0.80–1.10
**Start of the experiment, lamb aged 68 days (±3 days)**															
**C**	58.13 ± 3.12	31.54 ± 0.97	26.59 ± 2.84	10.88 ± 1.41	77.72 ± 5.53	4.18 ± 0.86	1.87 ± 0.59	110.4 ± 19.4	59.56 ± 23.21	900.75 ± 275.53	1.30 ± 0.24	0.27 ± 0.08	2.48 ± 0.12	2.78 ± 0.5	1.14 ± 0.11
**T1**	58.05 ± 2.38	32.07 ± 1.28	25.98 ± 2.96	10.01 ± 1.97	74.70 ± 7.46	3.96 ± 0.45	1.97 ± 1.15	139.23 ± 48.32	60.11 ± 19.52	892.3 ± 288.23	1.51 ± 0.39	0.34 ± 0.08	2.46 ± 0.24	3.00 ± 0.38	1.14 ± 0.14
**T2**	57.59 ± 5.07	32.88 ± 3.02	24.71 ± 2.95	10.37 ± 1.34	74.06 ± 8.61	4.21 ± 0.27	1.87 ± 0.87	108.26 ± 12.33	61.41 ± 21.74	466.87 ± 203.84	1.43 ± 0.47	0.30 ± 0.14	2.54 ± 0.20	3.22 ± 0.55	1.19 ± 0.11
***p*** **-values**	0.94	0.33	0.35	0.48	0.34	0.58	0.97	0.49	0.98	0.36	0.47	0.54	0.63	0.15	0.57
**End of the experiment, lamb aged 118 days (±3 days)**															
**C**	62.61 ± 1.99	30.54 ± 1.99	32.07 ± 2.50	7.64 ± 0.61	69.09 ± 5.48	4.68 ± 1.18	2.54 ± 0.27	126.89 ± 38.37	64.13 ± 7.68	460.39 ± 282.44	1.25 ± 0.35	0.29 ± 0.12	2.48 ± 0.09	2.88 ± 0.39	0.96 ± 0.07
**T1**	62.33 ± 2.79	31.74 ± 1.09	30.59 ± 2.65	6.92 ± 1.06	66.06 ± 7.03	4.19 ± 0.85	2.52 ± 0.64	162.80 ± 88.10	72.12 ± 10.64	308.67 ± 139.19	1.34 ± 0.14	0.28 ± 0.09	2.58 ± 0.08	2.98 ± 0.33	0.95 ± 0.07
**T2**	62.68 ± 3.44	31.67 ± 1.35	31.01 ± 2.46	7.41 ± 0.81	65.41 ± 4.23	4.06 ± 0.35	2.49 ± 0.32	140.06 ± 18.96	67.80 ± 11.03	381.51 ± 255.69	1.34 ± 0.23	0.34 ± 0.13	2.56 ± 0.14	2.96 ± 0.30	1.00 ± 0.07
***p*** **-values**	0.96	0.16	0.42	0.17	0.32	0.26	0.97	0.37	0.21	0.36	0.66	0.46	0.11	0.79	0.25

* Par.—parameter; Prot.—total protein; Alb.—albumin; Glob.—globulins; Creat.—creatinine; Gluc.—glucose; Bilir.—total bilirubin; AST—aspartate transferase; GGT—gamma-glutamyl transferase; CK—creatin kinase; Chol.—cholesterol; Trig.—triglycerides; Ca—calcium; P—phosphates; Mg—magnesium. ** C—group without added tannins; T1—group that received 9.46 g of tannin product/kg DM of the diet; T2—group that received 18.87 g of tannin product/kg DM of the diet. *** Ref.—Reference values.

## Data Availability

Data are contained within the article and throughout the Supplementary Materials.

## Supplementary Materials

The following supporting information can be downloaded at https://www.mdpi.com/article/10.3390/life14121556/s1. Figure S1: HPLC chromatograms of the most abundant compounds in Farmatan Plus^®^ recorded in the single ion recording mode.
