# Supplementary figures and images for: Characterization of Chestnut Tannins: Bioactive Compounds and Their Impact on Lamb Health
Source: Life (Basel). 2024 Nov 27;14(12):1556. doi: 10.3390/life14121556 (PMC11678802; doi:10.3390/life14121556)

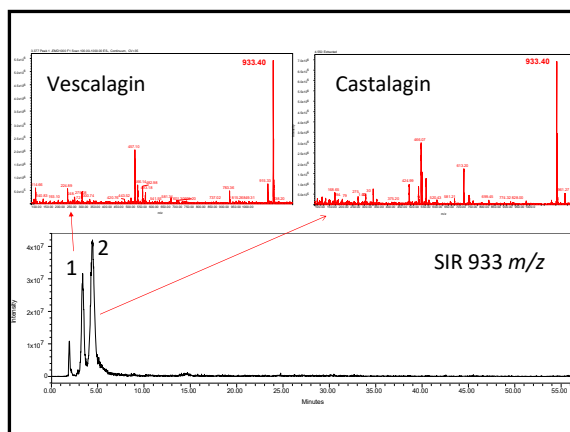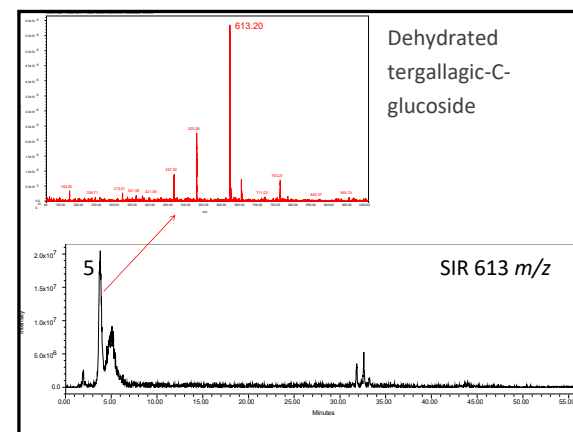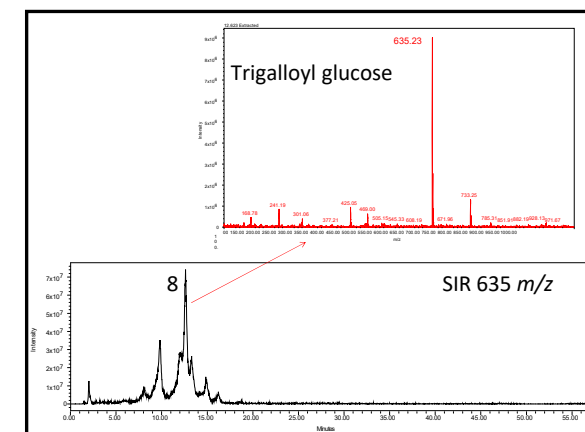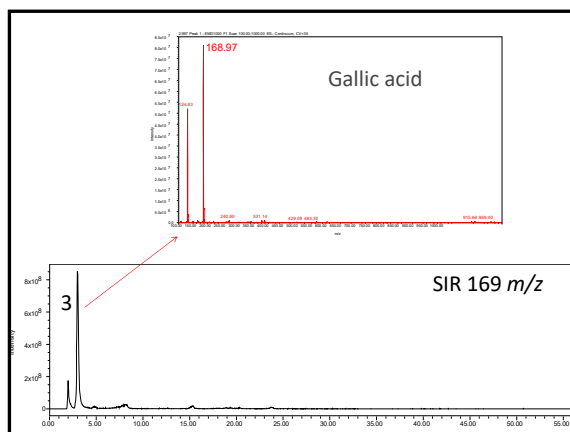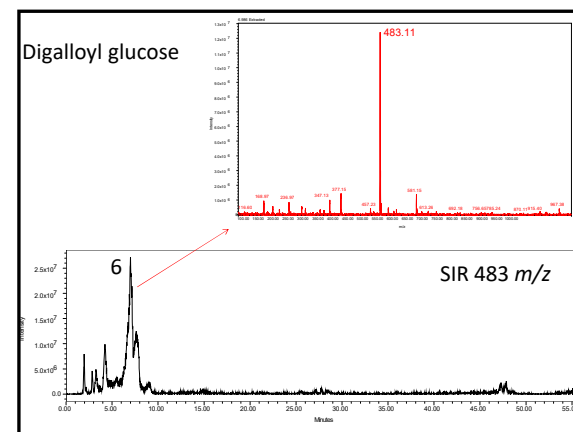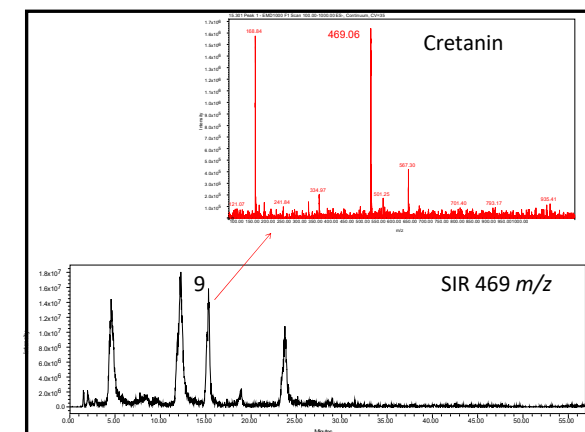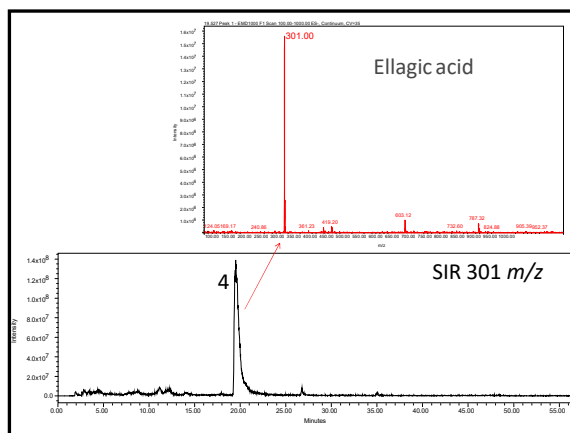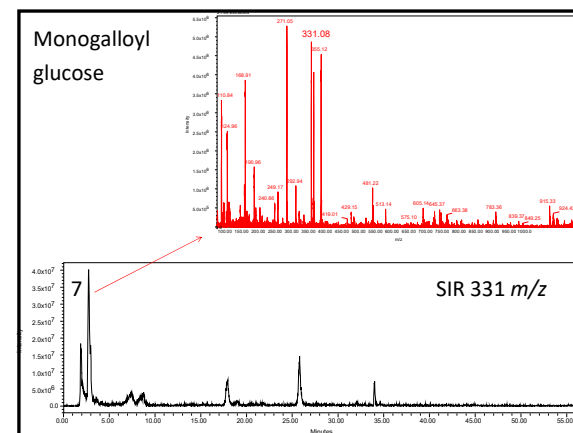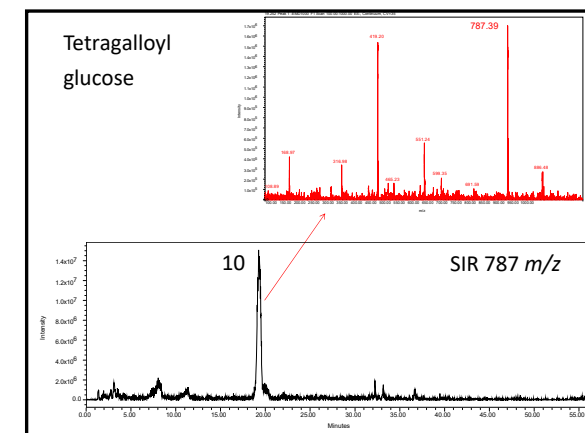

Supplement: Supplementary file 1 [file life-14-01556-s001.zip › life-3311038-supplementary.pdf]
